# Factors associated with blood culture sampling for adult acute care hospital patients with suspected severe infection: a scoping review using a socioecological framework

**DOI:** 10.1093/jacamr/dlaf043

**Published:** 2025-03-20

**Authors:** Deborah Bamber, Nicholas Fahy, Tim Coats, Clare Gillies, David R Jenkins, Eva M Krockow, Anthony Locke, Alison Prendiville, Laura Shallcross, Carolyn Tarrant

**Affiliations:** Department of Population Health Sciences, University of Leicester, Leicester LE1 7RH, UK; RAND Europe, Cambridge CB2 8BF, UK; Leicester Royal Infirmary, University Hospitals of Leicester NHS Trust, Leicester LE1 5WW, UK; Department of Population Health Sciences, University of Leicester, Leicester LE1 7RH, UK; Leicester Royal Infirmary, University Hospitals of Leicester NHS Trust, Leicester LE1 5WW, UK; School of Psychology and Vision Sciences, University of Leicester, Leicester LE1 7RH, UK; PPIE Representative, Leicester, UK; School of Design, University of the Arts London, London SE1 6SB, UK; Institute of Health Informatics, University College London, London WC1E 6BT, UK; Department of Population Health Sciences, University of Leicester, Leicester LE1 7RH, UK; National Institute for Health and Care Research (NIHR) Greater Manchester Patient Safety Research Collaboration (GM PSRC)

## Abstract

**Background:**

Reliable blood culture sampling for patients with suspected severe infection is critical, but evidence suggests that blood culture samples are not always reliably collected for acute hospital patients with severe infection. There is a pressing need to understand the barriers and facilitators of optimal sampling practices for patient safety and antimicrobial stewardship.

**Methods:**

We conducted a scoping review to identify evidence of factors associated with reliable blood culture sampling, for adult patients with suspected severe infection in acute care in high-income countries. We searched bibliographic databases (MEDLINE, Scopus, Web of Science, CINAHL), reference lists and citations between 2013 and February 2024. Findings were mapped to a socioecological framework.

**Results:**

We retrieved 1823 records from the database searches; 7 studies were eligible for inclusion, with 8 additional studies identified from reference lists and citation searches. All 15 included papers identified factors at the individual level of influence, including patient factors (demographics, clinical signs and symptoms) and staff factors (knowledge of guidelines, attitudes and beliefs, emotion, clinical experience and training, and perception of economic cost). Evidence gaps existed in relation to factors at interpersonal, situational, organizational, community and policy levels.

**Conclusions:**

Our review provides insights into blood culture sampling practices in hospitals, and highlights possible evidence gaps as potential areas to guide future research and inform the development of interventions to improve blood culture sampling in hospitals. Existing research has been dominated by a focus on individual levels of influence, with a paucity of evidence on influences at the interpersonal, situational, organization, community and policy levels.

## Introduction

Reliable blood culture sampling for patients with suspected severe infection is critical for improved patient safety, and to reduce antibiotic overuse. Blood cultures are the primary diagnostic test to detect and identify severe infection and susceptibility to antibiotics, providing vital information for clinical decision-making.^1^

National and international guidelines on the management of sepsis include the taking of blood cultures as a critical step.^2–5^ Reliable blood culture sampling is vital to inform the diagnostic process for patients with suspected severe infection, enabling initial prescribing decisions to be reviewed and revised based on the results of microbiological culture and antibiotic susceptibility testing.^6–8^ Blood culture results also play an important role in antimicrobial stewardship; they enable tailoring of antibiotic choice in hospitalized adults with severe infection^9^ (positive microbiology results informing the optimization of antibiotics) and can help in reducing use of unnecessary antibiotics in patients being treated ‘just in case’,^10^ when negative microbiology results are obtained. The presence of microbiology results for all patients with suspected severe infection has been identified as one of three priority factors with the greatest influence on antibiotic prescribing practice in hospitals,^5^ although results may only impact on management in a relatively limited number of patients.^11^ Evidence suggests, however, that blood culture samples are not reliably collected when indicated. Studies have found as few as a third of hospital patients with suspected severe infection having samples taken when antibiotics are started.^12–14^ Failure to collect blood culture samples contributes to sustaining broad-spectrum antibiotic overuse and prolonged courses of antibiotics, problems that continue to increase in hospitals.^15,16^

Recommendations and interventions have been developed for optimizing the blood culture pathway, from ordering and sample collection to transportation to the microbiology laboratory for testing; these have tended to focus specifically on clinical and technical good practice, such as skin preparation and sample volume, or quality indicators.^17–19^ Little attention has been paid to behavioural, social or organizational factors that may impact on blood culture practices, although evidence indicates that behavioural factors have an influence. An interview study with healthcare staff in three middle-income countries highlighted a varied and heterogeneous set of behavioural barriers and enablers to blood culture sampling, including lack of priority of blood cultures, perceived lack of value of blood cultures, staff perception of their role in ordering blood culture samples, the role of guidelines, and social norms.^20^

While a focus on behavioural barriers can inform the development of interventions targeting staff practice, blood culture sampling is best understood as a complex process involving multiple individuals, embedded in a wider social and organizational context. Blood culture request and collection requires coordination across individual staff members, and blood sampling practice is likely to be shaped by local organizational priorities and processes, and context-specific factors. As Broom *et al.*^21^ have argued, tackling complex problems in the field of antimicrobial use requires an awareness of multilevel influences; in their interview study of antibiotic use they identified social relationships (including perceptions of risk, hierarchies and inter- and intraprofessional dynamics) and institutional structures, such as population factors and resources, as having a strong influence on antibiotic prescribing. Similarly, we suggest that efforts to improve the reliability of blood culture sampling requires an understanding of the multidimensional barriers and facilitators of optimal practice.

To better understand reliable blood culture sampling practice in hospitals, we conducted a scoping review to systematically identify and present existing literature around the factors associated with reliable blood culture sampling in high-income countries. This was part of a wider study (NIHR156452) to optimize sampling at the pre-analytical stage and to co-design interventions to improve practice. We framed the review within an adapted socioecological model, informed by ecological systems theory,^22^ in recognition of blood culture sampling forming part of a complex system of relationships that occur across different levels of influence, at the individual, interpersonal, situational, organizational, community and policy levels. A similar conceptual framework was applied previously in the field of antibiotic use to understand determinants influencing antibiotic prescribing among emergency department (ED) physicians in Singapore.^23^

Scoping searches of Google Scholar, the Cochrane Database of Systematic Reviews, Joanna Briggs Institute (JBI) Evidence Synthesis and PROSPERO identified no existing or proposed reviews about factors associated with reliable blood culture sampling for adult acute care patients in high-income countries. A systematic review of barriers and enablers of blood culture sampling exists but was limited to low- and middle-income countries;^20^ the transferability of these findings to high-income settings is likely to be limited. The findings of our scoping review will contribute new knowledge and advance understandings about blood culture sampling for patient safety and antimicrobial stewardship.

## Review question

What individual, interpersonal, situational, organizational, community and policy factors are identified in the existing literature as being associated with blood culture sampling in adult acute care patients with suspected severe infection in high-income countries, and where are the gaps in the evidence?

## Methods

The scoping review was conducted according to the JBI methodology for scoping reviews^24^ and reported in line with the Preferred Reporting Items for Systematic Reviews and meta-Analyses extension for Scoping Reviews (PRISMA-ScR) checklist.^25^

### Eligibility criteria

The eligibility criteria (Table 1) were developed using the PCC framework of Population, Concept, and Context. Papers were included if: the population was adult patients with suspected severe infection; the concept was blood culture sampling, specifically factors associated with blood culture sampling at the pre-analytical stage; and the context was acute emergency care in high-income countries. Existing literature relating to any study design were included: primary qualitative, quantitative and mixed-methods studies reporting empirical data published in peer-reviewed journals.

**Table 1.** Summary of the eligibility criteria

**Inclusion criteria**	
Population	Adult patients >18 years old, with suspected severe infection
Concept	Blood culture sampling at the pre-analytical collection phase, predictors or factors associated with sampling
Context	Acute emergency acute care
Location	International
Study design	Quantitative, qualitative, mixed methods; primary empirical research reported in peer-reviewed journals
**Exclusion criteria**	
Population	Patients <18 years old, without suspected severe infection
Context	Not high income setting; reported on differences in rates between types of hospital departments or wards
Concept	Clinical or technical aspects of sampling (e.g. volume of blood, number of sets of bloods, contamination), positive test results, diagnostic stewardship, the analytic (processing) and post-analytic (management) phases
Study design	Case reports, literature reviews, recommendations for practice, guideline adherence, intervention evaluations

### Search strategy

The search strategy was developed iteratively with help from an experienced research librarian and using authors’ extensive experience of research into antibiotic overuse and antimicrobial stewardship, subject expertise in antibiotic use, clinical microbiology and emergency medicine, and patient and public experience of hospital care.

Existing articles known to the authors were used to identify initial keywords and phrases. Practice searches using the MEDLINE database helped refine search terms and set limitations. We searched four bibliographic databases (MEDLINE, Scopus, Web of Science, CINAHL) (see Table S1, available as Supplementary data at *JAC-AMR* Online, for the search strategy). Articles were limited to the English language and those published between 2013 and February 2024, to reflect current health service guidance and practice over the last decade. Reference lists and citations of all included articles were screened for additional relevant articles. Quality assessments of the included studies were not undertaken, in line with scoping review guidance.^26^

### Source selection

Identified citations were exported into an EndNote 20 (Clarivate Analytics, PA, USA) library for reference management, and duplicates were removed. Citation details were imported into Rayyan,^27^ freely available software, to support the screening process.

Articles were independently screened for inclusion against the eligibility criteria, by title and abstract by one reviewer (D.B.). A 10% subset were simultaneously independently screened by a second reviewer (C.T.) for consistency. The two reviewers met to discuss results and modify the eligibility criteria where required. Full texts of eligible articles were retrieved and independently screened by the first reviewer and a 10% sample screened by the second reviewer before comparing results and reaching agreement. Any articles that the first reviewer was unsure about were discussed with the second reviewer as the topic expert. For one study, where although the abstract was in English, the full-text article was in Spanish, Google Translate was used to translate it. Articles that did not meet the inclusion criteria were excluded from the review, and the reasons for exclusion were recorded. The PRISMA-ScR flow diagram (Figure S1) shows the screening and review process.

### Data charting and presentation

A bespoke data extraction form was used to extract and descriptively map data by key source information and the *a priori* levels of the socioecological framework (Table 2). The form was piloted using three studies and modified accordingly. Data were extracted by one reviewer (D.B.) and verified by the second reviewer (C.T.) for accuracy and completeness, and regular meetings between the two were held to discuss the process and for quality assurance purposes. Findings were presented and discussed at wider project team meetings.

**Table 2.** Draft data extraction form

**Key source information**
Author/year of publication/country of origin
Aims/research question
Population/sample size/context
Methodology/methods
**Key relevant findings**
Individual-level variables that impact on reliable sampling: staff, e.g. knowledge and skills; role and identity/ownership and responsibility; attitudes, priorities, beliefs about value of blood testing, and consequences
Potential explanatory mechanisms relating to individual-level staff variables (e.g. not just ‘lack of feedback’ but ‘lack of feedback about patient outcomes means that junior doctors don’t see the consequences of their actions, reducing motivation to ensure blood samples are taken reliably’)
Individual-level variables that impact on reliable sampling: patient, e.g. demographics; diagnosis; treatment history
Potential explanatory mechanisms relating to individual patient variables
Interpersonal-level variables that impact on reliable sampling: social influences, e.g. norms, influence of others, multidisciplinary working, hierarchies
Potential explanatory mechanisms relating to interpersonal-level variables
Situational-level variables that impact on reliable sampling: time of day; location (e.g. emergency department, acute medical ward); demand/busyness/lack of time
Potential explanatory mechanisms relating to situational-level variables
Organizational-level variables that impact on reliable sampling: environment and resources (e.g. equipment); policies and guidelines; systems (including IT, automated systems)
Potential explanatory mechanisms relating to organizational-level variables
Community-level variables that impact on reliable sampling: patient groups/media; external targets; influential bodies
Potential explanatory mechanisms relating to community-level variables
Policy-level variables that impact on reliable sampling: e.g. national guidelines; resource allocation; healthcare regulations
Potential explanatory mechanisms relating to policy-level variables

## Results

### Article screen and study selection

Database searches retrieved 1823 records, with 1286 records remaining after duplicate removal. Screening of titles and abstracts excluded 1215 records as not meeting the eligibility criteria, resulting in 71 papers for full-text review. Of these, a further 56 were excluded, resulting in 7 included papers. Eight papers were also identified and included from reference lists and citations searches, resulting in a total of 15 studies included in the review (Figure S1).

### Description of the charted literature

A full description of the included papers can be found in Table S2. Consistent with the eligibility criteria, the included papers were published between 2013 and February 2024. Studies were conducted in a range of countries, with most reporting findings from the USA^28–33^ and/or Germany.^32,34–36^ All studies included were conducted in the context of acute, emergency care in public teaching and research hospitals, in publicly funded or state-organized health systems and in high-income countries. Most studies were quantitative (*n* = 13), using survey, electronic health records, case review, cohort or cross-sectional designs, one qualitative using semistructured interviews, and one mixed-methods cross-sectional study using hospital records, laboratory data and survey.

Included papers reported on whether blood culture samples were taken, on the quality of practices related to sampling, or knowledge and attitudes about sampling. Seven studies^32,34–39^ reported on a patient population where blood culture sampling is indicated (e.g. patients with discharge diagnosis of sepsis^40^). Eight studies^28–31,33,41–43^ reported on a wider population (e.g. patients admitted with fever) where local guidance or norms may indicate blood culture sampling; these studies provided evidence of factors associated with variation in sampling practices. Six papers^32,33,35,36,38,43^ reported on a healthcare professional sample; these staff were all physicians, but three of these papers also included nurses,^33,36,38^ one also included phlebotomists^38^ and one also included hospital/laboratory directors and managers.^36^ This scoping review therefore reports on factors that are associated with the collection of blood culture samples when indicated, or of potential value.

### Mapping the literature to the socioecological framework

**Figure 1.**
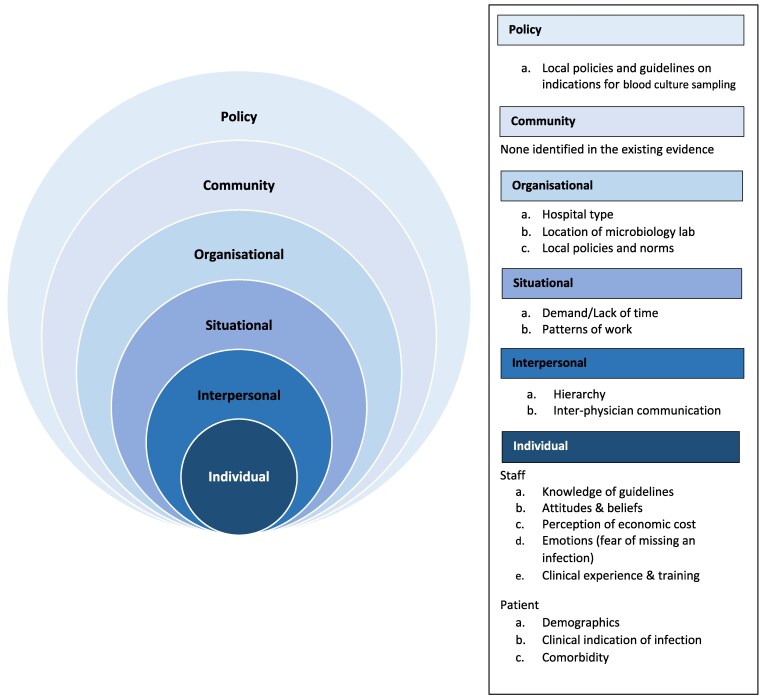
A socioecological framework of factors associated with reliable blood culture sampling for adult acute care patients with suspected severe infection.

Factors associated with blood culturd sampling were mapped to the socioecological framework. The factors are described here for each level as follows (Table 3), and represented in Figure 1.

**Table 3.** Factors associated with blood culture sampling mapped to a socioecological framework

Author, year	Individual level		Interpersonal level	Situational level	Organizational level	Community level	Policy level
	Patient	Staff					
Berninghausen, 2024	Sex; clinical presentation of infection; comorbidity	—	—	—	Hospital type	—	—
Choi, 2019	Clinical presentation of infection	—	—	—	—	—	—
Chotirmall, 2016	Sex; age; comorbidity	—	—	—	—	—	—
Dräger, 2022	Sex; age; clinical presentation of infection	Knowledge of guidelines	—	—	Hospital type	—	—
Dunne, 2015	SIRS	—	—	—	—	—	—
Fabre, 2018	Clinical presentation of infection	Knowledge of guidelines; attitudes & beliefs; clinical experience & training; emotions (fear of missing an infection)	Interphysician communication	—	Hospital type	—	Local policies and guidelines on indications for blood culture sampling
Foong, 2022	Clinical presentation of infection; SIRS	—	—	Time of day; day of week	—	—	—
Howard-Anderson, 2019	Clinical presentation of infection	Clinical experience & training	Interphysician communication	—	Hospital type	—	—
Linsenmeyer, 2016	Clinical presentation of infection	—	—	—	—	—	—
Martin-Sanchez, 2019	Age	—	—	—	—	—	—
Moema, 2018	Clinical presentation of infection; comorbidity	Knowledge of guidelines	—	—	—	—	—
Raupach-Rosin, 2017	Clinical presentation of infection	Knowledge of guidelines; attitudes & beliefs; perception of economic cost; clinical experience & training; job role	—	Lack of time	Onsite microbiology laboratory	—	—
Schmitz, 2013	Clinical presentation of infection; SIRS	Perception of economic cost	—	Lack of time	—	—	—
She, 2015	Clinical presentation of infection	Attitudes & beliefs; perception of economic cost and time to result	—	—	—	—	—
Sturkie, 2021	Ethnicity; clinical presentation of infection	—	—	—	—	—	—

### Individual level

All 15 papers reported on factors associated with blood culture sampling at the individual level of influence, either at a patient or healthcare staff level.

### Individual patient level

Individual patient-level factors were identified in all 15 papers; these included patient sex, age, ethnicity, clinical presentation and comorbidity.

Male patients were identified as more likely than female patients to have blood culture samples taken, or have adequate samples collected (defined as ≥2 sets before antibiotic therapy).^34,42,43^ Patients aged >60 years were identified as being more likely to have blood culture samples taken in two studies of undifferentiated emergency populations,^42,43^ and those aged >65 years being statistically more likely to have at least one sample taken than younger patients in a study of patients attending ED with severe infection.^39^ Ethnicity was a factor in one US study, which found a significant association between white ethnicity and the likelihood of having a sample taken.^31^

Most studies (*n* = 11/15) reported that a clinical presentation of suspected severe infection, either with a known or unknown focus of infection, was associated with reliable or adequate blood culture sampling.^28–36,38,41,43^ Indicators of clinical concern were: fever, increased body temperature (≥38°C), hypothermia; lower systolic blood pressure (≤100 mm Hg); chills; respiratory difficulties; rigor; and dizziness. Other indicators included a central line accompanied by a rise in C-reactive protein and abnormal white cell counts. Less sampling was associated with presence of a urinary catheter.^28^ The presence of systemic inflammatory response syndrome (SIRS—body temperature of >38°C or <36°C, heart rate of >90 beats/min, respiratory rate of >20 breaths/min^44^) was also identified as a factor increasing the likelihood of blood culture sample collection, collection of multiple sets of samples,^36,37^ or as a significant predictor for sample requests.^28^

In three studies,^34,38,42^ comorbidity and immunosuppression were also identified in patients as being associated with higher levels of blood culture sample collection.

### Individual staff level

Six papers^29,32,33,35,38,43^ reported on individual staff-level influences on blood culture sample collection; these included knowledge of guidelines, attitudes and beliefs about blood culture sampling, perceptions of cost, emotional factors and clinical experience.

Staff knowledge, or lack of knowledge, of guidelines was identified as influencing sampling.^33,35,38,43^ In studies that identified suboptimal blood culture sampling practice, survey data indicated that many healthcare staff had no or insufficient knowledge of guidelines, and few had ever been trained on them.^35,38^ A substantial number were unsure about requesting samples despite the existence of guidelines.^43^

Staff attitudes and beliefs about the importance of blood culture sampling in ruling in or out infection were associated with sampling.^32,33,35^ Sixty-seven percent of clinicians surveyed in one study rated blood culture sampling as acceptable in ruling in infection but only 36% thought that it could rule out infection.^32^ Beliefs about blood culture sampling identified as reasons for why samples were not taken were: sampling can be repeated when treatment fails; too many blood cultures are collected in daily practice; there are few local antibiotic resistances; an accurate result cannot always be expected; and they are unnecessary when the infection is known and appropriate antibiotics are given.^35,43^ Three papers found that staff perceived economic cost to be a reason as to why samples were not collected when indicated, or why staff valued them negatively,^32,35,36^ and one also cited perceived length of time to result as a factor that negatively impacted on blood culture collection.^32^ One study identified fear of missing an infection as a driver of blood culture sampling.^33^

Three papers reported on clinical experience and training.^29,33,35^ Junior (resident) doctors’ level of training was associated with the number of blood culture samples requested overnight,^29^ although no clear trend was identified. Recent experience of taking blood culture sample sets (within the last 30 days) was a predictor of compliance with good sampling practice (correct initiation and number of sets, fill and hygiene).^35^ Staff with fewer years of clinical experience were more likely to report concerns about missing infections in patients,^33^ driving oversampling by those who were less clinically experienced. Professional job role, however, was not identified as a predictor of sampling practice.^35^

### Interpersonal level

Only two studies reported on factors associated with blood culture sample collection at the interpersonal level: interphysician communication and hierarchy.^29,33^ In one cohort study with a population of hospitalized patients with fever, resident doctors were significantly more likely to request samples for patients with sign-out instructions (transfer of patient knowledge and plan) from a senior advising a ‘full fever work-up’.^29^ In a staff survey study, a small proportion of staff agreed that communication among prescribers, and between prescribers and nurses, would improve sampling practices.^33^

### Situational level

Three studies reported on factors associated with blood culture sample collection at the situational level.^28,35,36^ These included workload and time constraints as reasons for the non-collection of samples for patients with sepsis or where otherwise indicated.^35,36^ Night-shift work and working at the weekend were found to be associated with increased likelihood of sample requests for patients with at least one episode of abnormal body temperature.^28^

### Organizational level

Factors relating to blood culture sampling at the organizational level were identified in five papers;^29,33–35,43^ these included differences between hospital types, and the influence of local policies.

Two studies^29,34^ reported on differences between types of hospitals and the number of sets collected for corresponding patient groups: significantly more blood culture samples were collected at a tertiary hospital compared with a primary care hospital;^34^ and more tests were requested in an academic hospital compared with a community hospital.^29^ Staff reported being more likely to order blood cultures for patients with similar signs and symptoms in the context of ICU compared with non-ICU settings,^33,43^ and in university hospitals as opposed to non-university hospitals. One study found better blood culture practice in non-surgical departments, and in hospitals that had an onsite microbiology laboratory.^35^

### Policy level

Very limited evidence was identified in the literature about the impact of policy, regulations, resource allocation and guidelines on blood culture sampling. Some studies assessed adherence to national guidelines;^31,34,35,37^ one study included discussion of the possible impact of national guidelines on practice, but did not specifically assess this.^28^ A survey of healthcare staff identified a lack of clarity about indications for blood culture sampling, highlighting that local policies and practices requiring sampling for all patients with fever could contribute to overuse of blood cultures, and noting that ‘guidelines do not provide specific recommendations for when blood cultures should be drawn’. Around 70% of staff surveyed agreed that that a protocol with indications would improve blood culture ordering practices.^33^

## Discussion

This scoping review has identified from the existing literature factors associated with blood culture sampling for adult acute care patients with suspected severe infection. Using the socioecological framework to map these factors has provided insight into the multifaceted and complex nature of blood culture sampling practices in hospitals, and highlighted potential evidence gaps as areas for improvement and to inform future research.

The review demonstrates that existing research into influences on blood culture sampling is dominated by a focus on individual-level factors of influence. Evidence suggest that sampling practices are influenced by demographic and clinical features of patients, including clinical indicators of severe infection, and comorbidity. As might be expected, research also identifies aspects of staff knowledge, attitudes and beliefs as influences on sampling. Lack of knowledge about guidelines was identified as a key barrier to sampling, as was negative attitudes about the value and benefits of blood cultures. Lack of time was also cited as a barrier. These behavioural factors map onto established models of behaviour change,^45^ and indicate promising areas for interventions that target individual staff behaviour to optimize blood culture sampling.

Our use of the socioecological model has highlighted that other factors at multiple levels of influence have rarely been studied. There is scant evidence on the influence of interpersonal, situational and organizational influences. Influences at the community level have not been considered at all, and evidence of policy-level factors is very limited. It is possible that the focus on individual factors reflects the primary importance of these factors in shaping blood culture sampling practice; however, Suntornsut *et al.*’s^20^ systematic review of barriers and enablers of blood culture sampling in low- and middle-income countries also identified key barriers that existed at the levels of social influence and environmental context and resources. The little evidence that we found in this review highlighted significant multilevel influences on practice, which included the powerful influence of seniors on blood culture requesting practice, and the recognition of the importance of communication between staff. Social norms, hierarchy and peer influence have been identified as critical factors in relation to antibiotic use practices, and team culture and communication recognized as vital for antimicrobial stewardship. These issues merit further exploration in relation to blood culture sampling practices.^46–48^ The identified differences between healthcare organizations in the rates of blood culture sampling for similar patient groups (e.g. patients with sepsis) also suggests value in exploring wider organizational and contextual influences. Differences in sampling rates between healthcare organizations is well documented,^49,50^ but can be difficult to interpret in terms of comparability of patient populations, and further research is needed to explore how and why organizational factors shape practices. In particular, there is limited evidence on the impact of local policies, guidelines and norms for blood culture sampling, and this merits further attention.

Our review aimed to identify factors associated with reliable blood culture sampling for patients with suspected severe infection. This research, however, needs to be set in the context of the wider issue of diagnostic stewardship.^51^ While underuse of blood cultures is a significant problem, there is also evidence of widespread overtesting,^52^ i.e. requests for blood cultures when they are not indicated or may not be of clinical value. Problems arising from this overuse relate to cost and waste of scarce resources, but also, overtesting can drive the unnecessary use of antibiotics and contribute to the problem of antimicrobial resistance. One of the studies in our review identified influences on blood culture sampling practice that could be drivers of overuse,^33^ including fear of missing an infection, and local policies and practices promoting sampling for all patients with fever. Our review also identified uncertainty about when samples were indicated, despite the existence of guidelines, suggesting the need for further research into this area of uncertainty, and the potential value of algorithms and decision-support tools to aid the optimization of testing.^53,54^

To our knowledge, this is the first scoping review to identify and map the existing evidence on factors associated with blood culture sampling for acute care patients in high-income countries. The benefit of using the socioecological framework enabled us to understand the behavioural, social and organizational contexts in which sampling occurs, recognizing that sampling practices both shape and are shaped by such contexts.

The review had a deliberately broad focus, including research across a number of high-income countries, but the impact of contextual differences between the countries on blood culture practices was not explored, nor were differences between public and private healthcare providers. The review primarily focused on acute care settings, and transferability to other settings, such as outpatient clinics, is likely to be limited.

Several of the studies described here relied on self-reports of blood culture practices from surveys rather than direct observations. The extent to which conclusions can be drawn about the quality of practices is therefore limited. The purpose of this scoping review was not to critically assess the methodological quality of the evidence though, but instead to summarize the current evidence base and identify gaps in the literature. The review highlights the paucity of qualitative research in this area; only one of the identified papers used qualitative methods,^36^ but even these findings were presented in a quantitative manner. We suggest there is a pressing need for qualitative research to fully explore the diverse factors that shape blood culture sampling practice.

## Conclusions

Framing the review using a socioecological model highlights possible gaps within the research evidence, most notably, a paucity of evidence about multilevel influences on blood culture sampling. It also highlights the limitations of existing research into blood culture sampling, in terms of a lack of comparability of the patient populations included in studies, and the lack of qualitative research. Understanding the influences on reliable blood culture sampling, and setting these in the context of wider considerations of diagnostic stewardship, will be critical in informing the design of interventions to optimize blood culture sampling.

## Supplementary Material

dlaf043_Supplementary_Data

## Supplementary data

Figure S1 and Tables S1 to S2 are available as Supplementary data at *JAC-AMR* Online.
