# Correction to: Factors associated with blood culture sampling for adult acute care hospital patients with suspected severe infection: a scoping review using a socioecological framework

**DOI:** 10.1093/jacamr/dlaf133

**Published:** 2025-07-25

This is a correction to: Deborah Bamber, Nicholas Fahy, Tim Coats, Clare Gillies, David R Jenkins, Eva M Krockow, Anthony Locke, Alison Prendiville, Laura Shallcross, Carolyn Tarrant, Factors associated with blood culture sampling for adult acute care hospital patients with suspected severe infection: a scoping review using a socioecological framework, *JAC-Antimicrobial Resistance*, Volume 7, Issue 2, April 2025, https://doi.org/10.1093/jacamr/dlaf043

The following changes have been made to the originally published manuscript. A second affiliation has been added to details for co-author Carolyn Tarrant. The enumeration in the author list is altered to read: “[…and Carolyn Tarrant^1,8*^” instead of “[…]and Carolyn Tarrant^1*^”. The affiliations list has additionally: “^8^National Institute for Health and Care Research (NIHR) Greater Manchester Patient Safety Research Collaboration (GM PSRC)”.

These emendations have been made to the article

