# Supplementary material for: Factors associated with blood culture sampling for adult acute care hospital patients with suspected severe infection: a scoping review using a socioecological framework
Source: JAC Antimicrob Resist. 2025 Mar 20;7(2):dlaf043. doi: 10.1093/jacamr/dlaf043 (PMC11924178; doi:10.1093/jacamr/dlaf043)
Supplement: dlaf043_Supplementary_Data [file dlaf043_supplementary_data.zip › Supplementary Figure 1 PRISMA flow diagram for v2.docx]

**Identification of studies via other methods**

**Identification of studies via databases**

Records removed *before screening*:

Duplicate records removed (n = 537)

Records identified from:

Databases (n = 1,823)

(MEDLINE n = 401

SCOPUS n = 1,160

Web of Sciences n = 211

CINAHL n = 51)

**Identification**

Records screened

(n = 1,286)

Records excluded

(n = 1,215)

Reports not retrieved

(n = 0)

Reports sought for retrieval

(n = 13)

Reports sought for retrieval

(n = 71)

Reports not retrieved

(n = 0)

**Screening**

Reports assessed for eligibility

(n = 13)

Reports excluded:

Wrong population (n = 1)

Wrong source of evidence (n = 5)

Reports assessed for eligibility

(n = 71)

Reports excluded:

Wrong outcome (n = 64)

Studies included in review

(n = 15)

**Included**
