# Supplementary material for: Factors associated with blood culture sampling for adult acute care hospital patients with suspected severe infection: a scoping review using a socioecological framework
Source: JAC Antimicrob Resist. 2025 Mar 20;7(2):dlaf043. doi: 10.1093/jacamr/dlaf043 (PMC11924178; doi:10.1093/jacamr/dlaf043)
Supplement: dlaf043_Supplementary_Data [file dlaf043_supplementary_data.zip › Supplementary Table 1 and 2 for v2.docx]

Supplementary information Table 1: MEDLINE practice search strategy 19/02/2024 adapted as appropriate for each database search

| **Search** | **Query** | **Records retrieved** |
| --- | --- | --- |
| #1 | blood culture*.mp. or Blood Culture/ | 31163 |
| #2 | (sampl* or collect* or take or taking or practice*).mp. | 5481888 |
| #3 | exp Emergency Service, Hospital/ or acute care.mp or critical care.mp. or intensive care.mp. or admission ward*.mp. or emergency department*.mp. or emergency unit*.mp. or emergency ward*.mp. | 474941 |
| #4 | exp Adult/ or adult*.mp. | 8789931 |
| #5 | 1 and 2 and 3 and 4 | 691 |
| #6 | limit 5 to (english language and yr="2013 -Current") | 401 |

Supplementary Information Table 2: Description of the included studies

| **First author, year, country** | **Aim/ research question** | **Population & Setting** | **Methods** |
| --- | --- | --- | --- |
| Berninghausen 2024  Germany | The extent to which blood culture (BC) sampling was performed and what factors were associated with the absence of general or inadequate BC sampling | ED admitted patients with discharge diagnosis of sepsis.  3 emergency departments (ED) from a primary care hospital, a secondary care hospital, a tertiary care hospital | Quantitative: Retrospective cohort |
| Choi 2019  Singapore | To compare practices of obtaining BCs in ED and inpatient general wards and examine the clinical impact of such focus | Patients getting BC in ED (comparator was patients without BC on ED getting BC on ward <24 hrs).  Single university hospital | Quantitative: Retrospective comparative cohort |
| Chotirmall 2016 Ireland | To determine the relationship between urgent BC and mortality or length of stay | Acute emergency admissions.  All unselected emergency admissions.  Single tertiary care university hospital | Quantitative: Longitudinal cohort |
| Dräger 2022 Switzerland | To assess the appropriateness of BC collection according to local hospital guidelines and assess differences in knowledge and attitude of prescribers regarding BC ordering and collection | Patients presenting to ED, hospitalised in medical and surgical wards.  Internal medicine physicians, ED physicians and surgeons.  Single centre university hospital and a multicentre survey conducted in 3 hospitals (2 non-university and one university hospital) | Quantitative: Retrospective – hospital electronic health records and staff survey |
| Dunne 2015  UK | To evaluate the effect of BCs on clinical management in acute surgical admissions | Acute surgical admissions receiving IV antibiotics within 24 hours of admission.  Single hospital | Quantitative: Electronic health records and case note review |
| Fabre 2018 USA | To survey prescribers’ knowledge, attitudes, and perceptions regarding BC to help in developing future interventions aimed at optimising blood culture testing of adult patients | Providers of clinical inpatient care (medical and surgical physician assistants and nurse practitioners, medicine house staff, hospitalists, intensivists and infectious disease physicians). Single hospital | Quantitative: Survey |
| Foong 2022  USA | To determine the factors affecting BC ordering in febrile and hypothermic inpatients | Hospitalised adults ≥18 years with at least one episode of fever or hypothermia.  Single academic hospital | Quantitative: Retrospective |
| Howard-Anderson 2019 USA | To delineate what diagnostic tests overnight residents order in response to fevers and what sign-out, patient, and clinical factors influence ordering practices | Hospitalized adults with fever overnight (no standard definition of fever).  Resident doctors.  Two internal medicine teaching hospitals | Quantitative: Prospective cohort |
| Linsemeyer 2016  USA | To identify the physician-selected indication and yield of BCs ordered after hospitalisation to an acute medical service and to identify populations in which BCs may not be necessary | Hospitalised patients for whom BCs were ordered and received by the microbiology laboratory.  Single teaching hospital | Quantitative: Prospective cohort |
| Martin-Sanchez 2019 Spain | To determine the clinical profile and initial management of elderly patients with acute infections attending Spanish EDs, and to analyse whether there are any differences compared to younger adults. | Elderly patients (≥65 years) with acute infection in EDs.  Multicentre | Quantitative: Descriptive, cross-sectional – register data |
| Moema 2018  South Africa | To determine BC utilisation among healthcare workers in an ED, to quantify the contamination rates of BC, and to assess the knowledge and practices of healthcare workers | Patients admitted to ED and meeting definition for suspected BSI/sepsis.  Doctors, nurses, phlebotomists.  Single public-sector academic hospital | Mixed methods:  Cross-sectional study of medical records and lab data; staff survey |
| Raupach-Rosin 2017 Germany | To assess knowledge, attitudes and practice of physicians regarding BC diagnostics | Medical doctors, final year medical students | Quantitative: staff survey (based on qualitative focus groups) |
| Schmitz 2013  Italy, UK, France & Germany | To gain insights into current BC testing practices in intensive care units (ICU) | ICU directors, residents, nurses; microbiology laboratory directors, managers | Qualitative: Semi-structured interviews |
| She 2015  USA & Germany | To understand clinicians' perspectives on the diagnosis and management of patients with BSI and ascertain how new diagnostic tests would influence medical decisions and potentially patient care | Practitioners in infectious diseases/microbiology, critical care, internal medicine, haematology/oncology, general medicine | Quantitative: Survey |
| Sturkie 2021  USA | To measure frequency and yield of BC obtained, describe how often performed according to guideline indications, identify proportion of patients meeting sepsis criteria, and identify predictors of BC obtainment | Adult patients in observation department with suspected skin/soft tissue infection.  Single hospital | Quantitative: Retrospective cohort |
